# The role of defense styles and psychopathological symptoms on adherence to conspiracy theories during the COVID-19 pandemic

**DOI:** 10.1038/s41598-023-30531-0

**Published:** 2023-03-01

**Authors:** Francesca Gioia, Chiara Imperato, Valentina Boursier, Christian Franceschini, Adriano Schimmenti, Alessandro Musetti

**Affiliations:** 1 Department of Humanities, University of Naples Federico II, Naples, Italy; 2 Department of Humanities, Social Sciences and Cultural Industries, University of Parma, Parma, Italy; 3 Department of Medicine and Surgery, University of Parma, Parma, Italy; 4 Faculty of Human and Social Sciences, UKE-Kore University of Enna, Enna, Italy

**Keywords:** Psychology, Health care

## Abstract

Due to the unpredictability of the COVID-19 pandemic situation, individuals felt uncertain and insecure. As a consequence, conspiracy theories flourished and quickly spread. In the current study, we examine the relationship between general and COVID-19-related conspiracy theories, cognitive reflection, psychopathological symptoms, and defense styles in a sample of Italian adults. A total of 450 participants (50.2% male; mean age = 40.89 years, SD = 12.15) took part in an online survey. Two linear regression models on the general (explained variance 22.6%) and COVID-19-related (explained variance 33.0%) conspiracy theories have been tested. Among the predictive factors, older age, mania symptoms, and immature defenses facilitate adherence to conspiracy theories; on the opposite side, higher education, cognitive reflection, and mature defenses protected from adherence to conspiracy theories. The study provides some novel findings about factors that are significantly associated with general and COVID-19-related conspiracy theories, and highlights the pivotal role of individuals’ psychological defenses in conspiracy theories.

## Introduction

General conspiracy theories (CT) have been defined as attempts to explain uncommon and distressing events that are threatening or inconsistent with personal expectations, rejecting standard explanations and attributing the cause of these events to human malevolence instead^1–3^. Also, people who display monological belief systems may tend to overgeneralize a conspiracy theory to other events^4^; that is, the evidence supporting a conspiracy theory are not considered specific to that issue, but they are used to support a general pattern of beliefs^5^. This monological belief system provides automatic explanations for different and complex phenomena^4^, allowing individuals to deal with associated feelings of uncertainty and powerlessness^5^. As shown in research^1,6^, during crisis when people report feelings of threat or uncertainty^7,8^, they demonstrate stronger belief in CT. In this regard, the COVID-19 pandemic has represented an unprecedented global crisis, affecting individuals’ everyday life and mental health^9^. The risk of contamination and the repeated experiences of social distancing, isolation and restrictions due to quarantine worsened psychological distress and feelings of loneliness^10–12^. In this regard, during the COVID-19 pandemic, the online communication has been worldwide promoted^13^, but it also represented a “double-edged sword”^14^. On one side, online communication and interactions allowed to be safe and socially (although not physically) connected and informed; on the other side, the Internet fostered the dissemination of dysfunctional communication models and improper attempts to understand the ongoing distressing reality^15^. Indeed, online platforms and digital devices facilitated a high-speed diffusion of false and fake narratives^6,16^, fostering the so-called COVID-19 pandemic “infodemic” of misinformation, in which fake news dangerously spreads faster and more easily than the virus^17,18^. The unpredictability of the pandemic situation and the lack of a strong scientific and public consensus on communication strategies to cope with COVID-19 increased people’s mistrust and intolerance of uncertainty^19,20^. As a consequence, many individuals have turned to informal information sources, such as online platforms and social media where several CT have been developed and shared, highly interrelated with adherence to general CT^21,22^. For decades, numerous CT have emerged concerning scientific and social issues (e.g., water fluoridation, the vaccine/autism link, and the 5G mobile technology/COVID-19 link) have been materialized with terrible consequences for public health^23,24^. Similarly, during the COVID-19 pandemic, it was suggested that COVID-19 is a fake news, the virus spread occurred on purpose as a bioweapon, the governments are prolonging the emergency situation to pursue their anti-democratic objectives, the 5G technologies are causing or accelerating the COVID-19 spread and powerful individuals are trying to inject microchip quantum-dot spy software through the vaccine and control people^25^.

Problematically, during the prolonged pandemic condition characterized by high fear, low confidence, and low trust^26^, the COVID-19-related CT have fostered higher rejection of rational preventive measures, less adherence to governments’ guidelines and less willingness to be vaccinated due to less confidence in science and scientists^1–3,16^.

Overall, specific socio-demographic characteristics have been found to be associated with adherence to CT. In particular, younger individuals and being male were more likely to endorse CT^22^. Likely, younger individuals are more exposed to CT because of their larger use of social media^27^ and males tend to be more open to new ideas and experiences than women^21,27,28^, who instead are more prone to assume preventive health measures^29^. Moreover, higher levels of education seem to buffer the adherence to CT, perhaps because well-educated individuals might show stronger scientific knowledge and/or analytical or critical thinking skills^30^. Beyond socio-demographic characteristics, believing in CT is a combination of social (e.g., social exposure to CT, feelings of belonging to a group) and individual factors (e.g., personality traits, psychological risk factors)^1,30^.

Generally, most CT pry about emotions, intuitive thinking and gut reactions, whereas individuals who are more reflective are also more resistant to conspiratorial content^31,32^. As Stecula and Pickup^31^ suggested, cognitive reflection refers to the capacity to override gut reactions, discerning better between truth and falsities. According to the dual-process theories of cognition^31,33,34^, this reflective way of processing information is opposed to the intuitive mode, which is more spontaneous, immediate, and exposed to the risk of bias in information processing. The cognitive reflection is logical and calculating and requires effort, motivation, and concentration^31,33,34^, suggesting that falling for CT is a result of more intuitive and less reflective style of thinking^35^. Indeed, reflective individuals have been described as able to discern fake and real news and as responsible social media users^36,37^. Similarly, in the COVID-19 pandemic context, cognitive reflection helped individuals to refrain from incorrect intuitive answers on social media: evidence indeed shows that individuals who are high in cognitive reflection are less likely to endorse CT^31^.Concerning the well-known association between the adherence to CT and psychopathological symptoms, several studies highlighted that CT may represent a (maladaptive) coping mechanism for individuals with high levels of depression and anxiety or psychological distress leading to worse psychological conditions^30,38,39^. More specifically, CT seem to provide simplified, causal explanations for distressing situations, allowing individuals to regulate acute stress and negative feelings, and restoring perceived control over events and agency^40^. In contrast, denial of COVID-19 as a threat and refusal of vaccines motivated by CT might paradoxically allow individuals to tolerate worries and uncertainty by perceiving personal control over the situation, maladaptively refusing preventive measures and adherence to public health measures^1–3,16,39^. Furthermore, previous studies confirmed the association between beliefs in CT and psychosis or psychotic-like experiences^41,42^. Overall, several CT have no credible evidence and are based on inaccurate and frequently illogical thoughts. Psychotic experiences enhance the development of these types of thoughts and, in the COVID-19 pandemic context, psychotic psychological characteristics might lead to deeply irrational theories and engagement in magical thinking^41,43^. However, despite conspiratorial individuals generally show explicit or symptoms of mental disorders^38^, adherence to CT is not necessarily pathological^30,43^. Therefore, previous studies explored the role of other psychological factors^1^, including defense mechanisms that can protect people from internal and external threats by preserving their psychological and ego integrity^44,45^. During distressing situations, individuals’ responses might fall under one of the main categories of defense styles, i.e., mature, neurotic, and immature^44,46^.

Defense mechanisms are automatic psychological processes that protect an individual from unpleasant emotions and feelings and prevent awareness of internal or external danger and stress^47^. According to Vaillant’s^48^ hierarchical model of defense styles, defense mechanisms can be classified as mature, neurotic, or immature which are characterized by different degrees of reality distortion. Mature defense mechanisms (e.g., sublimation that represents a way of redirecting unacceptable desires into more socially acceptable outlets) allow individuals to emotionally process and modulate distress while they maintain engagement with reality. Neurotic defense mechanisms (e.g., intellectualization that refers to the excessive use of abstract thinking to minimize or control disturbing feelings) are less adaptive strategies to reduce distress altering painful mental contents without dramatically distorting external reality. Immature defense mechanism (e.g., autistic fantasy that refers to the tendency to use fantasy as a substitute for human relationships or problem solving) imply a rigid and excessive alternation of personal feelings and/or a severe distortion of external reality^49^. Previous research demonstrated that the neurotic and immature defense styles are associated with detrimental effects^50^, such as psychopathology and psychological problems^49,51–53^.

Defense mechanisms might play a pivotal role during the COVID-19 pandemic^44,54,55^, as these mechanisms arise when feelings, urges and thoughts are too painful to elaborate^45,56^. The assumption of mature defenses such as sublimation and humor rather than immature defenses such as projection and hypochondriasis can dramatically impact on mental health, especially during the COVID-19 pandemic, when defense mechanisms might be more immature than usually^56^. Indeed, the COVID-19 pandemic as a stressful and traumatic experience might lead to distressing and anxious feelings which in turn might conduct to a lower level of functioning and immature and neurotic defense mechanisms in which pandemic-related anxiety is temporarily alleviated by the alteration of painful mental contents and/or distortion of external reality^56,57^. Indeed, as Albarracín^45^ explained, individuals who are facing unpleasant feelings or problems generally attempt to apply heuristics. Following the need for knowledge, whether the desired level of confidence is not met, individuals are likely to analyze the information more effortfully, using deeper analytical reasoning processes, while, following the ego’s need for defense, if the heuristics satisfy the desired level of confidence, they are likely to formulate a judgment based on the heuristic, rationalizing a desired conclusion using rudimentary reasoning processes. In this way, CT are a form of support for the ego^45^.

In summary, according to previous findings^7,8^, critical events might lead individuals to refuse standard explanations, recurring to CT^5^. In this regard, the COVID-19 pandemic, as an unprecedented global crisis, worsened psychological distress^10–12^ and feelings of powerlessness and uncertainty, promoting the development and spread of several COVID-19-related CT^21,22^. Overall, some socio-demographic characteristics^29,30^ and cognitive reflection^31^ seem to buffer against CT, whereas psychopathological symptoms might promote the adherence to CT in order to alleviate distressing psychological conditions^30,38,39^. Further factors such as individual defense mechanisms (in particular neurotic and immature defenses) might significantly help to understand why some individuals turn to general and COVID-19-related CT^44,54,55^. Therefore, the present study aimed at examining the previously unexplored association among defense styles, general and COVID-19-related CT, and further factors including socio-demographic characteristics, cognitive reflection, psychopathological symptoms, in a community sample of Italian adults. Based on the above-mentioned literature, we expected that younger age, being male individuals and lower education level would foster adherence to general and COVID-19-related CT, as well as psychopathological symptoms and neurotic and immature defense styles. Furthermore, we expected that cognitive reflection and mature defenses would negatively predict adherence to both general and COVID-19-related CT.

## Results

### Sample socio-demographic characteristics

We first collected data from 3536 participants. Then, we randomly selected participants based on a stratified sampling method. Specifically, we divided the collected data into groups according to the age and gender reported in official Italian statistics (dati.istat.it) and selected random samples that we calculated from each age and gender group.

Analyses were performed on a total of 450 participants, aged 18–60 years (*M* = 40.89, *SD* = 12.15), that voluntarily took part in the survey. Of the total participants, 226 (50.2%) were male, and 434 (96.4%) of Italian origins. As for the ethnicity of the remaining non-Italian participants, 10 (2%) were European, 2 (0.4%) were African, 3 Latino-American (0.6%), and 1 (0.2%) was North American. Most participants were graduated in high school or even have higher education (*n* = 415, 92.3%) and were in a relationship (*n* = 312, 69.3%). With regard to occupational status, 330 (73.3%) participants were employees.

### Preliminary analysis

We computed Pearson’s correlations among variables, and point-biserial correlations between dichotomous variables (i.e., gender, COVID-19 positivity participant, COVID-19 positivity close, and COVID-19 positivity acquaintance) and the other variables. Descriptive statistics with means, standard deviations and bivariate correlations are presented in Table 1.

**Table 1:** Means, standard deviations and Pearson's bivariate correlations among variables (*n* = 450) and point-biserial correlation with dichotomous variables (i.e., gender, and COVID-related variables).

	M	SD	1	2	3	4	5	6	7	8	9	10	11	12	13	14	15	16	17	18	19
1. Gender (1 = m)			-																		
2. Age	40.89	12.15	−0.021	-																	
3. Education years	14.68	3.44	−0.186***	−0.043	-																
4. CP participant	0.23	0.42	0.061	−0.102*	−0.153**	-															
5. CP close	0.64	0.48	0.077	−0.049	−0.111*	0.377***	-														
6. CP acquaintance	0.91	0.28	−0.014	0.009	0.048	0.150**	0.364***	-													
7. Cognitive Reflection (0–6)	3.11	1.87	0.103*	−0.107*	0.231***	−0.122**	−0.144**	0.114*	-												
8. Depression (0–4)	1.40	0.95	−0.113*	−0.113*	−0.054	0.066	0.021	0.123**	−0.033	-											
9. Mania (0–4)	1.09	0.85	−0.024	−0.137**	−0.067	0.077	0.068	0.106*	−0.054	0.218***	-										
10. Anxiety (0–4)	1.10	0.89	−0.163***	−0.194***	−0.090	0.132**	0.127**	0.079	−0.170***	0.655***	0.360***	-									
11. Somatic symptoms (0–4)	0.84	0.93	−0.117*	−0.073	−0.140**	0.182***	0.050	−0.023	−0.176***	0.484***	0.227***	0.585***	-								
12. Psychosis (0–4)	0.07	0.66	0.005	−0.055	−0.149**	0.110*	0.068	−0.038	−0.112*	0.149**	0.142**	0.278***	0.248***	-							
13. Repetitive thoughts and behaviours (0–4)	0.50	0.78	−0.053	−0.111*	−0.138**	0.122**	−0.011	−0.105*	−0.106*	0.480***	0.203***	0.555***	0.474***	0.363***	-						
14. Personality Functioning (0–4)	0.89	1.00	−0.084	−0.214***	−0.085	0.130**	0.002	−0.039	−0.038	0.598***	0.209***	0.552***	0.519***	0.274***	0.597***	-					
15. Substance use (0–4)	0.69	0.89	0.133**	−0.106*	−0.196***	0.162***	0.098*	−0.070	−0.142**	0.150**	0.102*	0.162***	0.185***	0.242***	0.308***	0.336***	-				
16. Mature defence style (1–9)	5.08	1.28	−0.097*	0.078	0.184***	−0.116*	−0.217***	−0.108*	0.056	−0.081	0.059	−0.054	−0.024	0.028	−0.038	−0.071	−0.049	-			
17. Neurotic defence style (1–9)	4.37	1.32	−0.236***	−0.088	0.021	0.009	−0.101*	−0.075	−0.018	0.091	0.223***	0.149**	0.180***	0.225***	0.186***	0.157***	−0.009	0.496***	-		
18. Immature defence style (1–9)	3.95	0.98	0.022	−0.066	−0.225***	0.178***	0.021	−0.045	−0.118*	0.273***	0.135**	0.281***	0.366***	0.332***	0.376***	0.413***	0.272***	0.187***	0.441***	-	
19. General CT (0–10)	5.46	2.39	0.012	0.035	−0.203***	0.128**	0.116*	0.130**	−0.173***	0.188***	0.231***	0.233***	0.214****	0.164***	0.192***	0.216***	0.127**	−0.109*	0.069	0.357***	-
20. COVID-related CT (6–42)	17.10	7.86	0.124**	0.140**	−.371***	0.185***	0.171***	0.047	−.302***	0.077	0.144**	0.165***	0.244***	0.171***	0.153**	0.139**	0.149**	−0.141**	0.015	0.368***	0.602***

*CP participant* COVID-19 positivity participants, *CP close* COVID-19 positivity of someone close, *CP acquaintance* COVID-19 positivity of acquaintance.

*p < 0.05; **p < 0.01; ***p < 0.001.

### Regression models

Analyses were performed using IBM SPSS v28. In order to explore the predictive effect of socio-demographic characteristics, cognitive reflection, psychopathological symptoms and defence mechanisms on general and COVID-19-related CT, we performed two hierarchical regression models, given that such statistical technique allows to introduce additional variables into the model to help to determine if the relationships are genuine^58^. Specifically, in the first model on general conspiracy we included socio-demographic characteristics (i.e., gender, age, and education years) on Step 1, cognitive reflection on Step 2, psychopathological symptoms (i.e., depression, mania, anxiety, somatic symptoms, psychosis, repetitive thoughts and behaviours, personality functioning, and substance use) on Step 3 and defence styles (i.e., mature, neurotic, and immature) on Step 4. In the second model on COVID-19-related CT, we included socio-demographic characteristics on Step 1, COVID-related variables (i.e., positivity of the participants, positivity of someone close to the participants and positivity of an acquaintance of the participants) on Step 2, cognitive reflection on Step 3, psychopathological symptoms on Step 4 and defence styles on Step 5.

Given that, we included variables belonging to the same measurement (i.e., depression, mania, anxiety, somatic symptoms, psychosis, repetitive thoughts and behaviours, personality and substance use were all Level-1 dimensions, whereas mature, neurotic and immature defence style were all dimensions of DSQ-40) and related each other, we firstly tested for multi-collinearity among these variables. Results suggested that there were correlations between such predictors, but not severe enough to cause suppression or confounding effect (tolerance ranging from 0.379 to 0.997; VIF ranging from 1.003 to 2.641)^59^. In addition, the data met the assumption of independent errors (general CT model: Durbin–Watson value = 1.925; COVID-19-related CT model: Durbin–Watson value = 2.004). Concerning data distribution, we considered normally distributed skew value <|2| and kurtosis value <|7|. All variables were normally distributed, except for psychosis (skewness = 3.59, kurtosis = 15.76)^60^. Therefore, we computed the IDF transformation on such variable^61^. Finally, the analysis of box-plots reveals three outliers, so we excluded such three cases from regression models.

Results of both hierarchical regression models on general and COVID-19-related CT were presented in Tables 2 and 3, respectively. As far as the model on general CT is concerned, in Step 1 only education was identified as a statistically significant predictor in the model, which explained 4.1% of variance. Adding the cognitive reflection variable, the explained variance increased to 5.7% and cognitive reflection was found to be a statistically significant predictor. A higher score of cognitive reflection was statistically predictive of lower levels of general CT. When it comes to the psychopathological symptoms, the explained variance increased to 14.6% and only mania was a statistically significant predictor. Higher levels of mania were predictive for higher levels of general CT. Lastly, the explained variance increased to 22.6% when adding defense styles. Both mature and immature defense styles were found to significantly predict general CT, while no relations were found with neurotic defense style. Specifically, high levels of mature defense style were predictive of lower levels of general CT, whereas the opposite relation was found with immature defense style.

**Table 2:** Results of hierarchical regression analysis for general CT (n = 447).

Predictor	B	SE	Beta	95%CI	F	R2 changes
Step 1					6.286***	0.041
Gender (1 = m)	−0.118	0.226	−0.025	−0.561, 0.326		
Age	0.004	0.009	0.022	−0.014, 0.022		
Education	−0.142	0.033	−0.203***	−0.207, −0.077		
Step 2					7.359**	0.016
Gender	−0.029	0.226	−0.006	−0.474, 0.416		
Age	0.002	0.009	0.010	−0.016, 0.020		
Education	−0.119	0.034	−0.171***	−0.186, −0.053		
Cognitive reflection	−0.166	0.061	−0.131**	−0.287, −0.046		
Step 3					5.708***	0.090
Gender	0.112	0.223	0.024	−0.327, 0.551		
Age	0.017	0.009	0.085	−0.001, 0.035		
Education	−0.094	0.033	−0.135**	−0.159, −0.029		
Cognitive reflection	−0.130	0.061	−0.102*	−0.249, −0.010		
Depression	0.085	0.161	0.034	−0.232, 0.401		
Mania	0.494	0.134	0.175***	0.230, 0.758		
Anxiety	0.135	0.190	0.050	−0.238, 0.509		
Somatic symptoms	0.094	0.150	0.036	−0.202, 0.389		
Psychosis	0.191	0.178	0.052	−0.159, 0.541		
Repetitive thoughts and behaviours	−0.007	0.192	−0.002	−0.385, 0.371		
Personality functioning	0.257	0.157	0.107	−0.051, 0.566		
Substance use	0.040	0.134	0.015	−0.223, 0.304		
Step 4					14.756***	0.080
Gender	−0.047	0.221	−0.010	−0.481, 0.388		
Age	0.018	0.009	0.094*	0.001, 0.036		
Education	−0.046	0.033	−0.066	−0.110, 0.018		
Cognitive reflection	−0.107	0.058	−0.084	−0.222, 0.007		
Depression	0.025	0.154	0.010	−0.279, 0.328		
Mania	0.550	0.131	0.195***	0.293, 0.806		
Anxiety	0.175	0.182	0.065	−0.183, 0.532		
Somatic symptoms	−0.017	0.145	−0.006	−0.302, 0.269		
Psychosis	0.056	0.175	0.015	−0.288, 0.399		
Repetitive thoughts and behaviours	−0.074	0.185	−0.023	−0.437, 0.289		
Personality functioning	0.068	0.153	0.028	−0.232, 0.368		
Substance use	−0.041	0.130	−0.015	−0.295, 0.214		
Mature defence style	−0.274	0.095	−0.147**	−0.462, −0.087		
Neurotic defence style	−0.115	0.104	−0.063	−0.319, 0.090		
Immature defence style	0.837	0.135	0.339***	0.571, 1.102		

Statistically significant predictors in bold.

*p < 0.05; **p < 0.01; ***p < 0.001.

**Table 3:** Results of hierarchical regression analysis for COVID-19 related CT (*n* = 447).

Predictor	B	SE	Beta	95%CI	F	R2 changes
Step 1					24.735***	0.143
Gender (1 = m)	1.095	0.693	0.071	−0.267, 2.457		
Age	0.078	0.028	0.123**	0.023, 0.133		
Education	−0.759	0.102	−0.334***	−0.959, −0.559		
Step 2					5.448**	0.031
Gender	0.950	0.684	0.061	−0.395, 2.295		
Age	0.088	0.028	0.139**	0.034, 0.143		
Education	−0.702	0.102	−0.309***	−0.902, −0.502		
CP participant	2.059	0.877	0.111*	0.336, 3.782		
CP close	1.525	0.800	0.095	−0.046, 3.097		
CP acquaintance	0.570	1.278	0.021	-1.942, 3.082		
Step 3					23.615***	0.042
Gender	1.483	0.676	0.096*	0.154, 2.812		
Age	0.072	0.027	0.114**	0.019, 0.126		
Education	−0.596	0.102	−0.263***	−0.796, −0.396		
CP participant	1.762	0.857	0.095*	0.077, 3.446		
CP close	0.900	0.790	0.056	−0.653, 2.454		
CP acquaintance	1.689	1.268	0.062	−0.802, 4.180		
Cognitive reflection	−0.912	0.188	−0.220***	-1.281, −0.543		
Step 4					2.833**	0.039
Gender	1.852	0.683	0.120**	0.509, 3.196		
Age	0.095	0.028	0.150***	0.040, 0.150		
Education	−0.548	0.101	−0.241***	−0.746, −0.349		
CP participant	1.191	0.859	0.064	−0.496, 2.879		
CP close	0.891	0.787	0.055	−0.655, 2.437		
CP acquaintance	1.774	1.284	0.065	−0.749, 4.298		
Cognitive reflection	−0.813	0.189	−0.196***	-1.184, −0.441		
Depression	−0.666	0.498	−0.081	-1.645, 0.313		
Mania	0.678	0.410	0.074	−0.129, 1.484		
Anxiety	0.221	0.585	0.025	−0.929, 1.371		
Somatic symptoms	1.097	0.462	0.129*	0.189, 2.004		
Psychosis	0.716	0.543	0.060	−0.352, 1.783		
Repetitive thoughts and behaviours	−0.279	0.591	−0.027	-1.441, 0.883		
Personality functioning	0.697	0.480	0.089	−0.246, 1.640		
Substance use	−0.140	0.410	−0.016	−0.946, 0.666		
Step 5					15.775***	0.074
Gender	1.376	0.673	0.089*	0.053, 2.700		
Age	0.098	0.027	0.154***	0.045, 0.151		
Education	−0.404	0.099	−0.178***	−0.599, −0.208		
CP participant	0.622	0.822	0.033	−0.995, 2.238		
CP close	0.677	0.758	0.042	−0.813, 2.168		
CP acquaintance	1.404	1.226	0.051	-1.005, 3.813		
Cognitive reflection	−0.751	0.180	−0.181***	-1.105, −0.396		
Depression	−0.852	0.476	−0.104	-1.786, 0.083		
Mania	0.873	0.399	0.095*	0.089, 1.657		
Anxiety	0.391	0.559	0.045	−0.707, 1.489		
Somatic symptoms	0.776	0.444	0.091	−0.096, 1.647		
Psychosis	0.303	0.531	0.026	−0.741, 1.347		
Repetitive thoughts and behaviours	−0.527	0.566	−0.051	-1.639, 0.585		
Personality functioning	0.082	0.466	0.010	−0.834, 0.997		
Substance use	−0.373	0.395	−0.042	-1.150, 0.403		
Mature defence style	−0.815	0.295	−0.134**	-1.394, −0.236		
Neurotic defence style	−0.407	0.316	−0.069	-1.027, 0.214		
Immature defence style	2.687	0.413	0.345***	1.875, 3.498		

Statistically significant predictors in bold.

*CP participant* COVID-19 positivity participants, *CP close* COVID-19 positivity of someone close, *CP acquaintance* COVID-19 positivity of acquaintance.

*p < 0.05; **p < 0.01; ***p < 0.001.

As far as COVID-19-related CT is concerned, age and education years were found to be statistically significant predictors in Step 1, while no relations were found with gender. Altogether socio-demographic characteristics explained 14.3% of variance. When it comes to COVID-19-related variables, having been positive for COVID-19 was a statistically significant predictor, therefore not having had COVID-19 was predictive of high levels of COVID-19-related CT. Adding COVID-19-related variables, the explained variance increased to 17.4%. Furthermore, cognitive reflection proved to be a significant predictor for COVID-19-related CT, so that high levels of cognitive reflection were predictive for lower levels of COVID-19-related CT and predictors included at this step explained 21.6% of variance. Adding psychopathological symptoms, only somatic symptoms were significant predictors of COVID-19-related CT. Specifically, high levels of somatic symptoms were predictive of high levels of COVID-19-related CT. Adding psychopathological symptoms, the explained variance increased to 25.5%. Lastly, both mature and immature defense styles were found to be significant predictor of COVID-19-related CT: high levels of mature defense style were predictive of low levels of COVID-19-related CT and high levels of immature defense style were predictive of high levels of COVID-19-related CT. Adding defense styles, the explained variance increased to 33.0%.

## Discussion

The present study contributes to the research field in relation to the predictive effect of the reflective cognition, psychopathological symptoms and the still understudied defense styles on general and COVID-19-related CT. According to previous findings, COVID-19-related CT negatively co-occurred with younger age and lower educational level^1^, whereas general CT positively correlated with psychopathological symptoms, such as anxiety and depression^16,22^. Our results support the view that monological belief systems lie behind CT: in fact, general and COVID-19-related CT were positively and strongly correlated, suggesting that some individuals might tend to overgeneralize a conspiracy theory to different, and even unrelated, phenomena^4^. Furthermore, the reflective cognition was negatively correlated with the adherence to both general and COVID-19-related CT^31,34,35^. Finally, consistent with the psychodynamic perspective^45,55^, mature defense style negatively co-occurred and immature defense style positively correlated with both general and COVID-19-related CT.

The linear regression models partially confirmed the study hypotheses. More specifically, differently from previous findings, in the current study older people adhered to both general and COVID-19-related CT more than younger individuals, who likely better understand the actual situation of COVID-19 and recognize misinformation on social media thanks to the global mobilization to boot young people’s media and digital literacy. Concerning gender differences, males appeared more prone to adhere to CT on COVID-19^21,27,28^; moreover, as previously highlighted in research^2,21,30–32^, more educated people showed more complex thoughts and cognitive reflection, testing knowledge claims for their validity and avoiding simplistic explanations (as CT) for a complex event as the COVID-19 pandemic. Furthermore, according to Stecula and Pickup^31^, people with higher level of cognitive reflection appeared more resistant to CT and better equipped to resist the intuitive gut reactions that conspiracies appeal to, discerning between fake and real COVID-19 pandemic-related news . Concerning the psychopathological symptoms, only mania symptoms positively predicted both general and COVID-19-related CT. Mania symptoms have been described as an intense manifestation of emotion dysregulation which might lead to difficulties in realty understanding, up to failures in reality testing and reality distortion^62^. We did not find a significant association to adherence to (COVID-19-related) CT and internalizing psychopathology, which is similar to other inconclusive studies^38,63,64^, suggesting that additional research is needed to clarify the links between CT and anxiety or depression. Finally, concerning the defense styles, mature and immature defense styles negatively and positively, respectively, predicted both general and COVID-19-related CT. Concerning general CT, they might support the ego, promoting the use of immature defense mechanisms to face unpleasant feelings and experiences^45^. Similarly, as previously stated^56,57^, stressful and traumatic experiences, as the ongoing COVID-19 pandemic, can lead to various psychopathological symptoms (especially anxious symptoms) frequently associated to neurotic and immature defense mechanisms (e.g., acting out, somatization, devaluation, projection), leading to severe alteration of painful mental contents and/or radical distortion of reality to temporarily alleviate them. Perhaps, the lack of control in stressful conditions as the COVID-19 pandemic might lead to or increase dysregulated thoughts and emotions In this regard, Schimmenti et al.^7^ highlighted the need to promote emotion regulation strategies during pandemic, helping individuals to better manage negative emotions and reduce the related risk of activating primitive defenses, such as denial or acting out.

Some limitations of the present study also need to be pointed out. Firstly, the use of self-report measures implies well-known potential method biases ranging from a misunderstanding of the study’s purposes to social desirability. Secondly, the participants involved in the study came from a specific (Italian) cultural context and the cross-sectional nature of the study does not allow causality inferences for the involved variables. Thirdly, the substance use subscale of the DSM-XC and the neurotic defenses subscale of the DSQ-40 showed weak internal reliability, though in line with other studies^65^. This calls for caution in the generalization of findings and suggests the opportunity to include clinician-reported measures in subsequent research to improve the reliability of the results. Furthermore, the low explained variance of the models could be due to the limited number of variables examined in this study, if compared to the tremendously complex etiology of general and COVID-19-related CT. Other factors, such as social characteristics, religious beliefs, clinical diagnosis of psychiatric disorders and prolonged exposure to COVID-19-related stressful conditions^30^ may have affected our results. In addition, we used scales to measure our variables, and given that it is known that every scale measures somewhat incorrectly, such kind of measurement may have affected the results. Lastly, the influence of CT in the current pandemic emergency cannot be underestimated. As previously highlighted^1–3^, higher adherence to COVID-19-related CT corresponded to lower willingness to follow public health recommendations and government guidelines and lower trust in science and scientists. Further studies on non-adherence to COVID-19 recommended measures as a serious public health issue are needed^2^. In this regard, longitudinal studies are essential to evaluate the long-term effects of adherence to CT. Future research should consider the role of defense styles as pivotal factors for preparation for possible future difficulties and emotional reactions to them^56^.

Notwithstanding these drawbacks, our study showed that some factors might represent common risk and protective factors for both general and COVID-19-related CT. Among the predictive factors, age, mania symptoms, and immature defense style seem to facilitate the adherence to CT, suggesting that experiences of crisis might enhance individuals’ lower level of functioning and immature defenses leading to distorted experiences of the external reality^49,56^. On the contrary, a less impulsive evaluation of the reality through higher education level, cognitive reflection, and mature defense style allow to better discern between truth and falsities, protecting from a more intuitive thinking and CT^31,33–35,55,66,67^.

The present study’s findings provided some novel and previously unreported issues. Different defense styles were significantly related to the adherence to general and COVID-19-related CT^1^. More specifically, assuming mature defenses rather than immature or neurotic defenses can make an enormous difference in mental health^55^. According to Marčinko et al.^56^, mature defense mechanisms contribute to improve resilience during crisis, making a significant difference in mental health. For example, humor as mature defense mechanism could enhance resilience during COVID-19 crisis^56^. Interestingly, adherence to CT does not necessarily seem to be just an expression of a lower education level, poor cognitive reflection, or psychopathological conditions. Instead, the adherence to CT seems to be mainly related to more deep and automatic psychological processes as defense mechanisms motivating the use of analytical or rudimentary reasoning processes.

The present findings shed new light on individuals that adhere to CT. These individuals are not inevitably affected by psychiatric disorders; rather, some of them seem to have difficulties in emotion regulation. Thus, as previously stated, psychosocial support and preventive interventions should be focused on awareness and management of emotions, in order to prevent the adherence to CT. Furthermore, the type of defense style used by people, especially during a global health crisis, needs to be monitored because a mature defense style allows people to emotionally process and modulate distressing conditions, maintaining engagement with reality, and using deeper analytical thinking^44,45,54,55^. In this regard, according to Gori et al.^68^, the involvement of mental health professionals appears necessary for reducing the risks for public health, and the circulation of Internet and media-based fake news, myths, and CT^66^, as these professionals should be able to identify the individuals’ defense style in play, to implement their insight and mentalization, and to promote the use of more mature and adaptative coping mechanisms for counteracting COVID-19-related dysfunctional responses, such as lack of cognitive reflection and manic symptoms.

## Methods

### Procedure

Data were collected from general population following a snowball sampling method from December 1, 2020 to May 8, 2022. Inclusion criteria were (a) being 18 years or older, (b) Italian native language, and (c) living in Italy during the COVID-19 outbreak. Participants were requested to complete a 20-min anonymous online survey on Google Forms. The online survey was promoted through social media. Furthermore, respondents were asked to spread the survey in their own network. All participants signed the informed electronic consent after obtaining general information about the aim of the study. Participation was confidential and voluntary, and all participants could withdraw from the study at any time. The Ethics Committee of the Center for Research and Psychological Intervention (CERIP) of the University of Messina approved this study (prot. No. 119094). The study was conducted in accordance with the Declaration of Helsinki and the ethical guidelines for psychological research laid down by the Italian Psychological Association (AIP). No remunerative rewards were given to participants.

### Measures

#### Socio-demographic characteristics

In this section information about gender, age, and educational attainments have been collected.

#### COVID-19-related exposure

Three dichotomous (yes/no) items were used to assess participant’s direct and indirect exposure to COVID-19 (i.e., “*Have you ever been infected by COVID-19?*”, “*Has anyone close to you ever been infected by COVID-19?*”, “*Have any of your acquaintances ever been infected by COVID-19*?”)*.*

#### Cognitive reflection test-long (CRT-L)

The Italian version of CRT-L^69^ (original version by Frederick, 2005^70^) has been used to evaluate participants’ ability to resist intuitive responses and to use an effortful reasoning. The CRT-L includes six open-ended problems (e.g., “*If it takes 5 min for five machines to make five widgets, how long would it take for 100 machines to make 100 widgets?*” for which the typical erroneous heuristic response is “*100 min*”, whereas the right response is “*5 min*”). The total score of CRT-L has been obtained by summing the number of correct responses. In the current study, Cronbach’s α was 0.77.

#### DSM-5 self-rated level 1 cross-cutting symptom measure (DSM-XC)

The DSM-XC^71^ (original version by American Psychiatric Association, 2013^72^) is a brief mental health assessment comprising 23 items across 13 trans-diagnostic domains of psychopathology. In the current study, eight of the 13 domains have been assessed: depression, mania, anxiety, somatic symptoms, psychosis, repetitive thoughts and behaviors, personality functioning, and substance use (e.g., “*Feeling down, depressed, or hopeless?*”, “*Avoiding situations that make you anxious?*”). Participants indicated how much (or how often) they have been affected by each symptom in the prior two weeks using a 5-point response scale from 0 (*none)* to 4 (*severe*). A score of 2 or higher in most domains, except substance use (score of 1 or higher), suggests clinically relevant mental health problems. The Cronbach’s α coefficients of anxiety and substance use were 0.72 and 0.56, respectively. For the two items dimensions, the Pearson’s *r* coefficients were 0.66 (depression), 0.30 (mania), 0.53 (somatic symptoms), 0.38 (psychosis), 0.56 (repetitive thoughts and behaviors), and 0.64 (personality functioning), always with *p* value < 0.001.

#### Defense Style Questionnaire-40 (DSQ-40)

The DSQ-40^65^ (original version by Andrews et al., 1993^73^) evaluates the individual defensive functioning according to three defense styles: (i) mature defenses, including sublimation, humor, anticipation and suppression (8 items, e.g. “*I’m able to keep a problem out of my mind until I have time to deal with it*”), (ii) neurotic defenses, including undoing, pseudoaltruism, idealization and reaction formation (8 items, e.g., “*If I have an aggressive thought, I feel the need to do something to compensate for it*”), and (iii) immature defenses, including projection, passive aggression, acting out, isolation, devaluation, autistic fantasy, denial, displacement, dissociation, splitting, rationalization, somatization (24 items, e.g., “*I often act impulsively when something is bothering me*”). The items were rated on a 9-point Likert scale from 1 (*Strongly disagree*) to 9 (*Strongly agree*). Scores on single mechanism defense have been calculated by averaging the two items measuring each defense, whereas scores on defense styles have been calculated through the mean of the items loading on each style. Elevated scores indicate higher use of the target defense/style. In the current study, the Cronbach’s α values were 0.65, 0.59, and 0.79 for mature style, neurotic style and immature style, respectively.

#### Conspiracy Mentality Questionnaire (CMQ)

The CMQ^74^ is a 5-item measure evaluating generic CT on an 11-point scale ranging from *certainly not* (0%) to *certain* (100%) (e.g., “*I think that government agencies closely monitor all citizens*”, “*I think that there are secret organizations that greatly influence political decisions*”). In the current study, the Cronbach’s α value was 0.87.

#### COVID-19 conspiracy theories

The 9-item of the COVID-19 conspiracy beliefs scale^75^ has been used to evaluate the accordance with the most widespread virus-related CT (i.e., the involvement of Bill Gates, the virus creation in a laboratory). The items were rated on a 7-point Likert scale from 1 (*Strongly disagree*) to 5 (*Strongly agree*). Higher scores strongly indicated CT. With the permission of the authors of the original scale, the COVID-19 conspiracy beliefs measure was translated from English to Italian using a back-translation method. One translator translated the measures from the source language (English) to Italian. A second and independent translator translated the new versions of the tests back to the source language. The original and the back-translated versions of the measures were then compared, and judgments were made about their equivalence. In the present study, Cronbach’s α coefficient was 0.86.

### Data analysis

Descriptive statistics and Pearson’s *r* correlations between the study variables were performed. Linear regression analyses were performed to explore the predictive effect of socio-demographic characteristics, cognitive reflection, psychopathological symptoms and defense mechanisms on general and COVID-19-related CT. All statistical analyses were performed using the Statistical Package for Social Sciences SPSS (Version 28 for Windows).

## Data Availability

The data that support the findings of this study are available on request from the corresponding author, AM. The data are not publicly available due to their containing information that could compromise the privacy of research participants.
